# Modified Dufourmentel Flap With Connective Tissue Grafting for Predictable Gingival Cleft Management: A Case Report

**DOI:** 10.1002/ccr3.70960

**Published:** 2025-09-22

**Authors:** Ruchi Harish, Saravanan Sampoornam Pape Reddy, Shaili Pradhan

**Affiliations:** ^1^ Department of Periodontology Army Dental Centre (Research & Referral) New Delhi India; ^2^ Department of Periodontology& Oral Implantology Kathmandu Medical College Public Limited Kathmandu Nepal

**Keywords:** case report, cleft, connective tissue, flap, gingival recession, microsurgical

## Abstract

Presence of a gingival cleft and marginal tissue recession promotes microbial invasion into tissues by plaque accumulation, leading to inflammation; thus, reducing the stability of marginal tissues. Hence, it is crucial to effectively control these gingival defects before they progress into more intricate mucogingival problems. This is a case report of a 39‐year‐old male patient who presented with the primary complaint of sensitivity in his upper right posterior teeth while brushing for the past 3 months. A two‐stage approach using ‘modified Dufourmentel flap’ with a microsurgical approach and connective tissue grafting was employed to address the white gingival cleft and associated gingival recession. The objective of the procedure was to eliminate the cleft completely and simultaneously achieve root coverage. Two years follow‐up revealed 100% recession coverage and cleft elimination as well as an increase in the width of attached gingiva from 1 to 3 mm and an increase in the width of the keratinized tissue from 2 to 4 mm, while the marginal gingiva remained stable. Employing a microsurgical modified Dufourmentel flap technique substantially enhanced the reliability of outcomes without causing any morbidity to the patient. Modified Dufourmentel flap with connective tissue grafting being a microsurgical procedure achieved complete cleft elimination, complete recession coverage, and enhanced keratinized tissue and attached gingiva two years postoperatively. These findings demonstrate the long‐term stability of this newly proposed technique.

**Summary**

- The modified Dufourmentel flap combined with connective tissue grafting provides a reliable and minimally invasive solution for managing gingival clefts and associated recession, achieving complete cleft elimination, enhanced keratinized tissue, and long‐term stability, as evidenced by successful outcomes maintained over two years.

## 1 Introduction

Marginal tissue recession (MTR) is a common mucogingival deformity characterized by displacement of the gingival margin apical to the cementoenamel junction (CEJ). Nevertheless, the existence of gingival clefts might not be detected at their early stage. Gingival clefts can be categorized as either “complete” or “incomplete” based on their extension into the keratinized tissue. They can be further be classified as “red” or “white” depending on the level of destruction to the underlying connective tissues. The term “white cleft” refers to a condition where the underlying connective tissue is completely destroyed, while the term “red cleft” is used to describe a condition where the underlying connective tissue is partially retained [1]. These terminologies are not included in the most recent classification of periodontal diseases by the American Academy of Periodontology and European Federation of Periodontology, despite their frequent occurrence [2]. Nevertheless, it is imperative to address these conditions as they can contribute to gradual loss of attachment and, when combined with reduced width of attached gingiva, accumulate bacterial plaque, thereby initiating a detrimental vicious cycle of tissue breakdown. Gingival cleft management requires a thorough assessment of various factors, including the nature and type of the cleft, clinical attachment loss, width of attached gingiva, width of keratinized tissue, and soft tissue phenotype [3]. In this case, a microsurgical strategy is implemented to address the white gingival cleft and recession coverage. It is composed of a unique flap configuration, a modified Dufourmentel flap, that is performed using an operating microscope with a 5× resolution.

## 2 Case History/Examination

**FIGURE 1:**
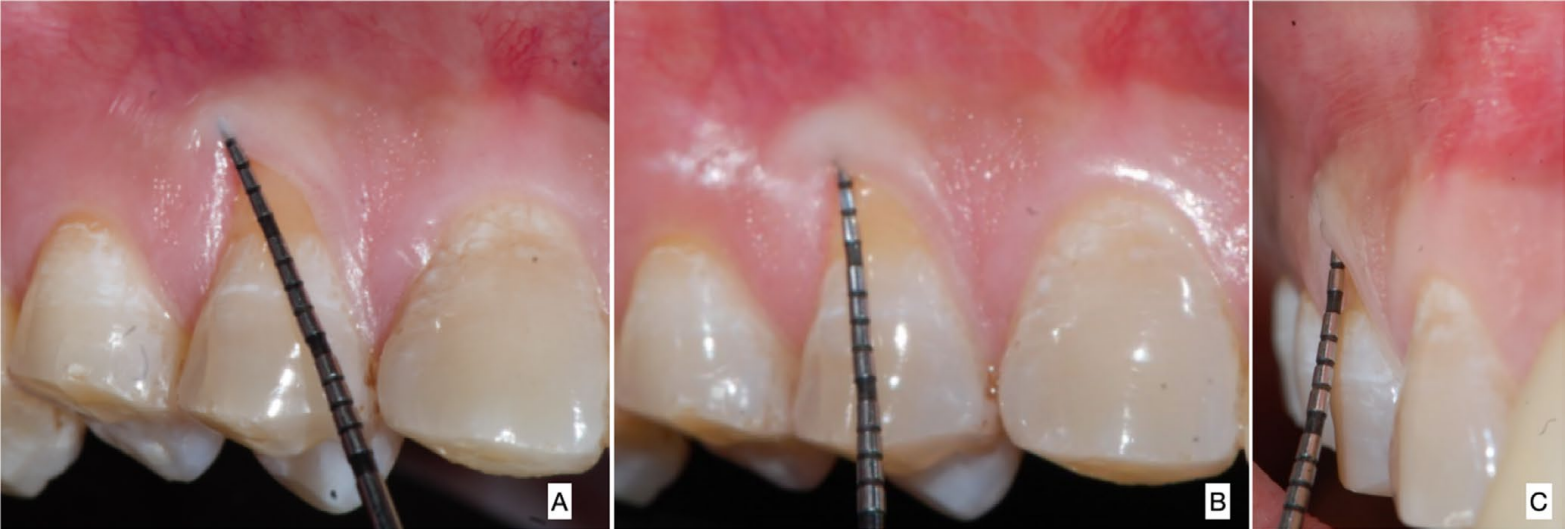
(A) Pre‐operative photograph showing the extension of gingival cleft, (B) pre‐operative photograph showing the probing depth and recession height, (C) pre‐operative photograph showing the proximal view.

This case is reported in accordance with CARE—Case REport 2017 guidelines [4]. A 39‐year‐old male reported experiencing sensitivity in his upper right posterior teeth while brushing for the past 3 months. In light of the patient's non‐contributory medical history, an ASA‐I physical status was assigned. The patient presented with moderate dental fluorosis and gingival bleeding in relation to the right first premolar14 (FDI Notation). At 5× magnification, examination confirmed the presence of a 2 mm white gingival cleft (Figure 1A). Additionally, the site exhibited MTR, which measured 3 mm in height (RH), 3 mm in width (RW), and exhibited 5 mm clinical attachment loss (CAL) (Figure 1B,C). The width of attached gingiva (WAG) measured 1 mm, while the keratinized tissue width (KTW) measured 2 mm. The soft tissue exhibited a thick phenotype, and the adjacent interdental papillae were intact in addition to the absence of step deformity. Based on clinical findings, a definitive diagnosis of Cairo's Class I A (−) MTR with a thick soft tissue phenotype in tooth no. 14 was arrived. After obtaining written informed consent, the patient was treated at a tertiary care postgraduate teaching institution, and all surgical steps were performed under 5× magnification utilizing an operating microscope (GPDM 80 Dental Operating Microscope, Gippon Inc., New Delhi, India.).

## 3 Methods (Differential Diagnosis, Investigations and Treatment)

**FIGURE 2:**
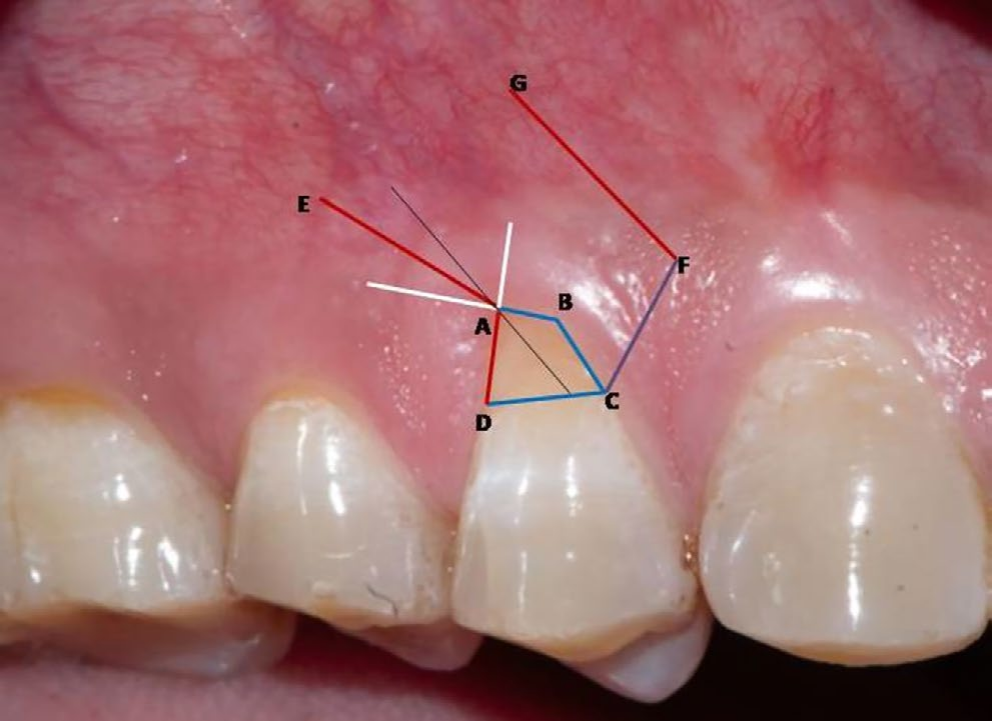
Clinical geometrical design of Dufourmentel flap.

**FIGURE 3:**
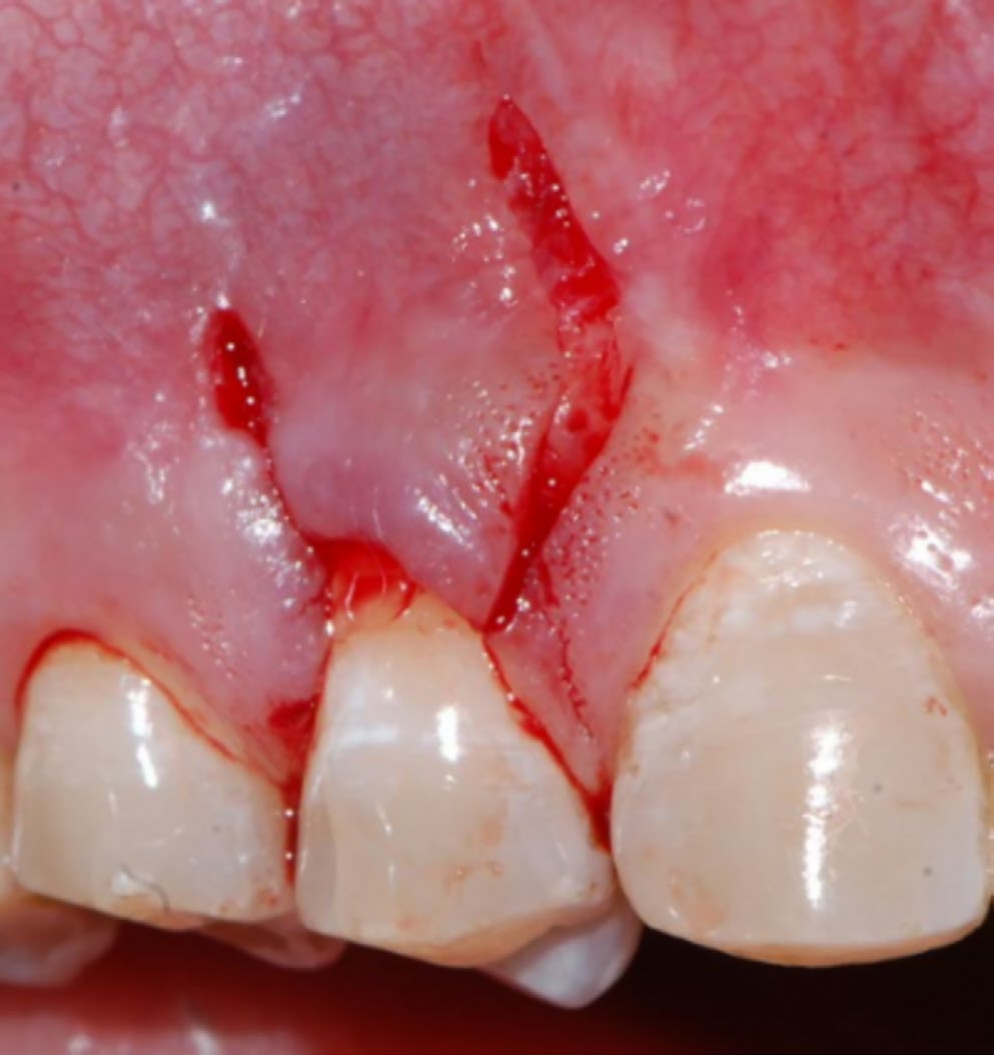
Intraoperative Dufourmentel flap design.

**FIGURE 4:**
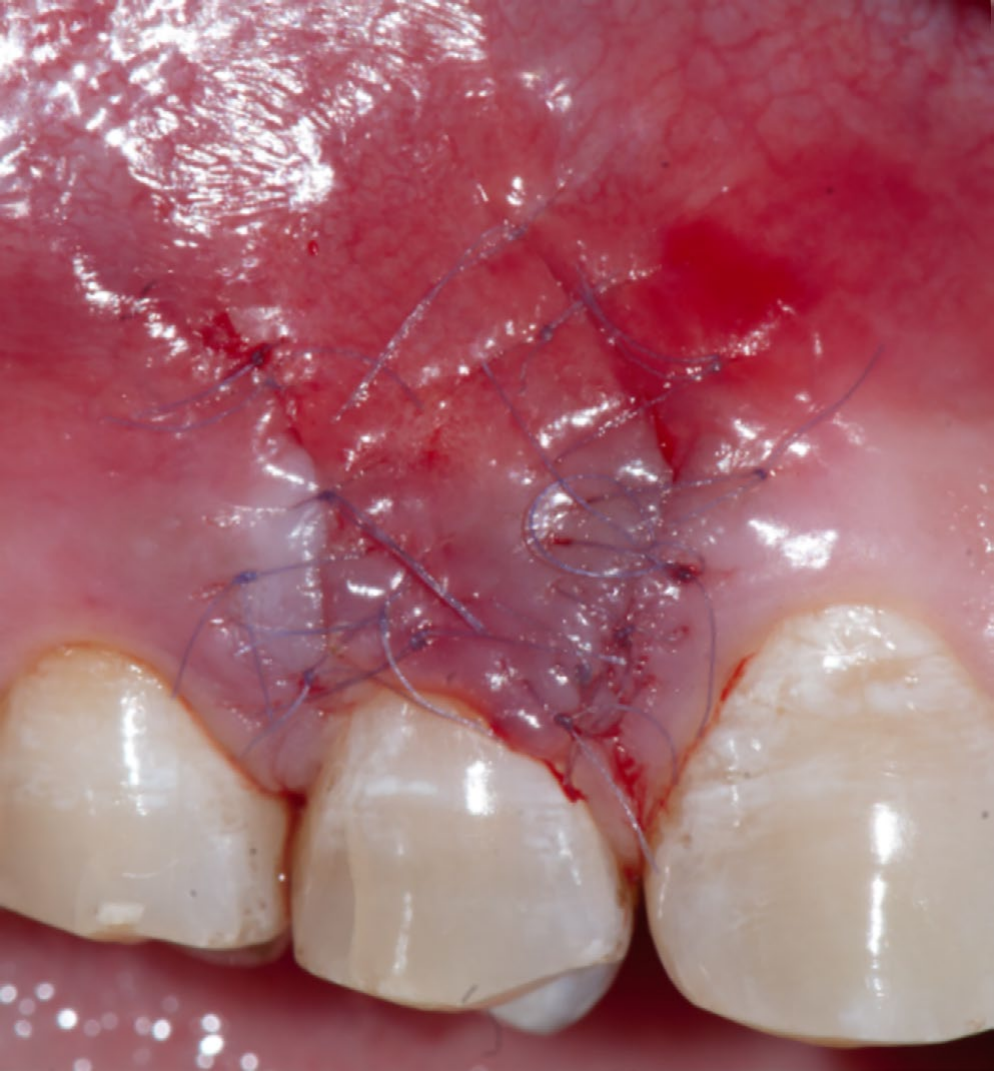
Flap suturing.

**FIGURE 5:**
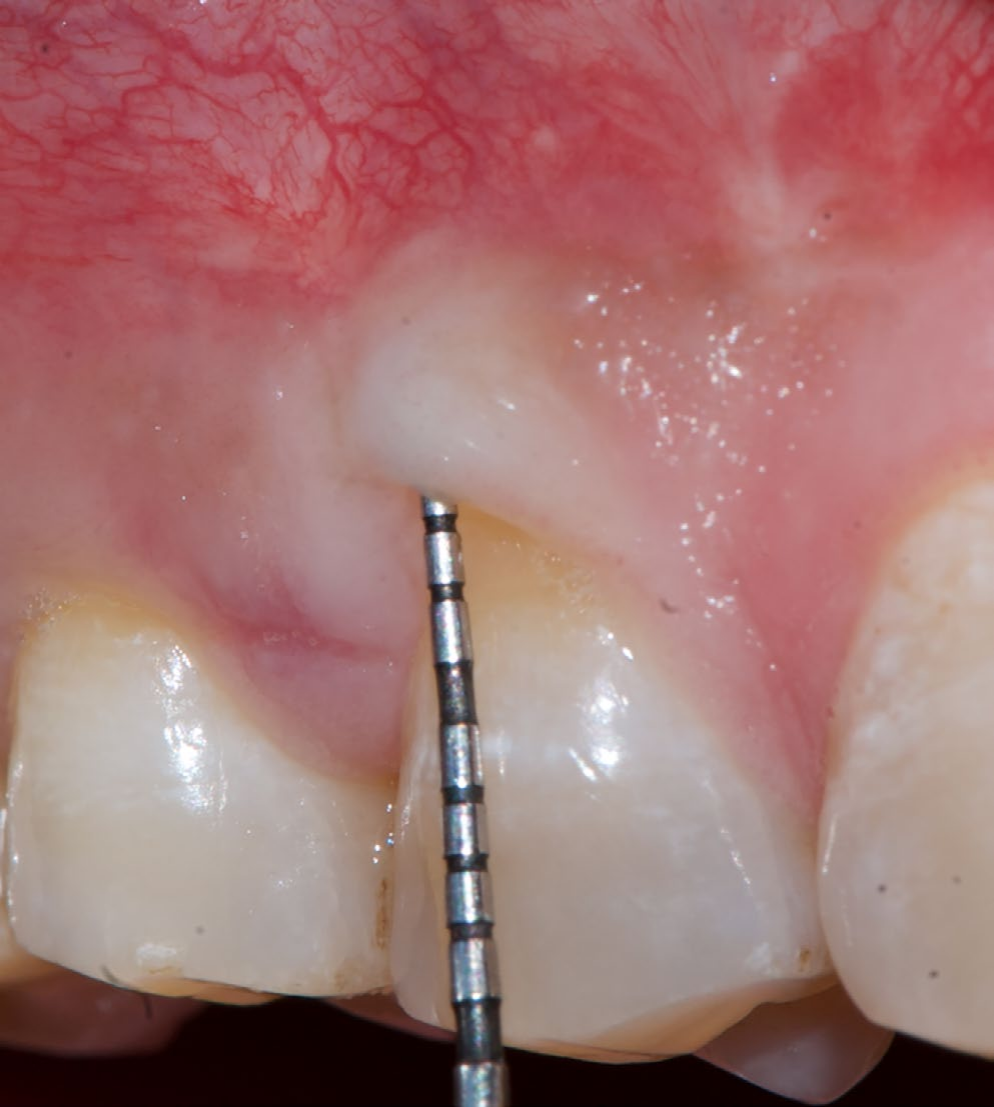
Residual recession of 1.5 mm and thick soft tissue phenotype.

**FIGURE 6:**
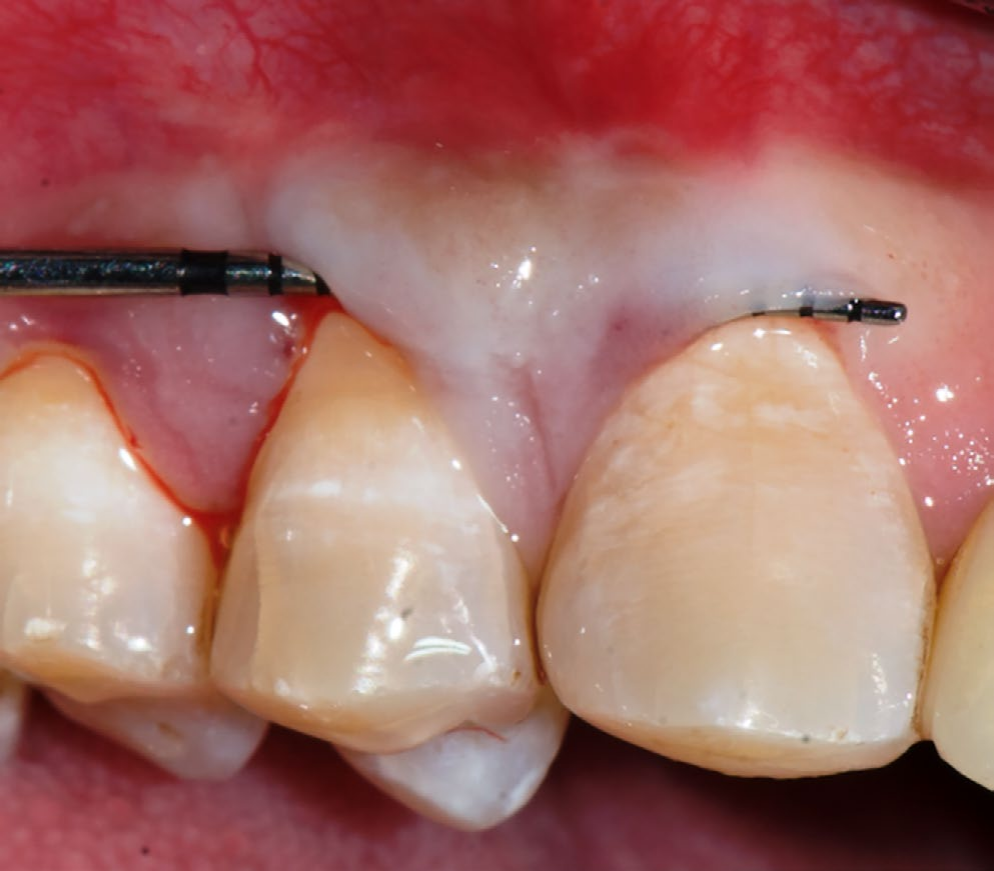
Preparation of supraperiosteal tunnel.

**FIGURE 7:**
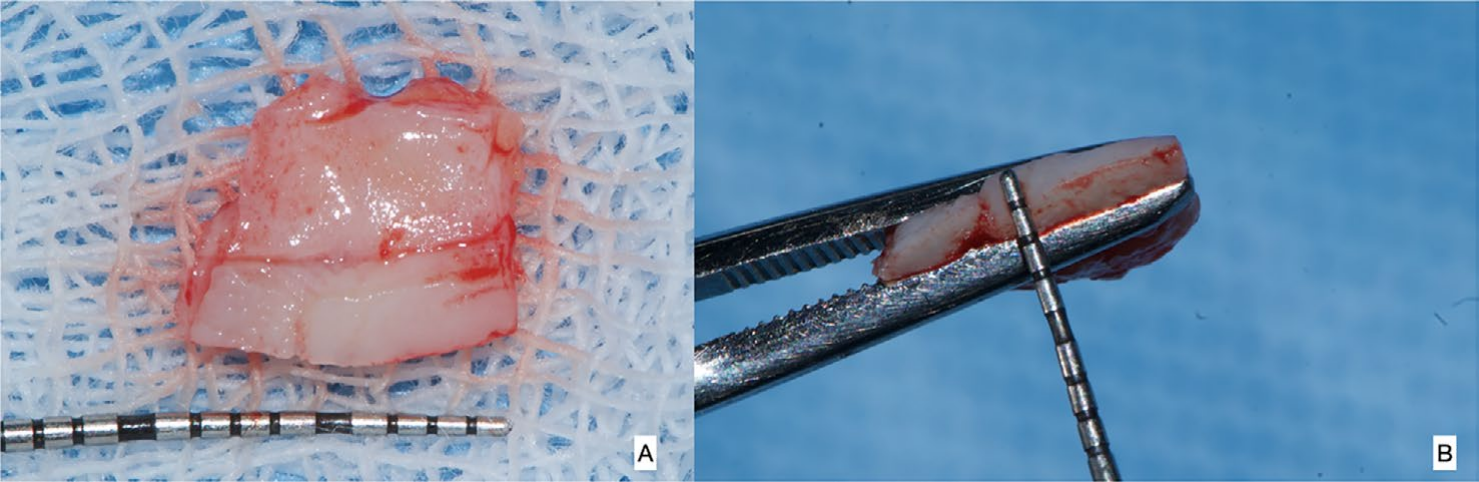
(A) Harvested connective tissue graft with epithelial collar, (B) thickness of CTG with epithelial collar of 2 mm.

**FIGURE 8:**
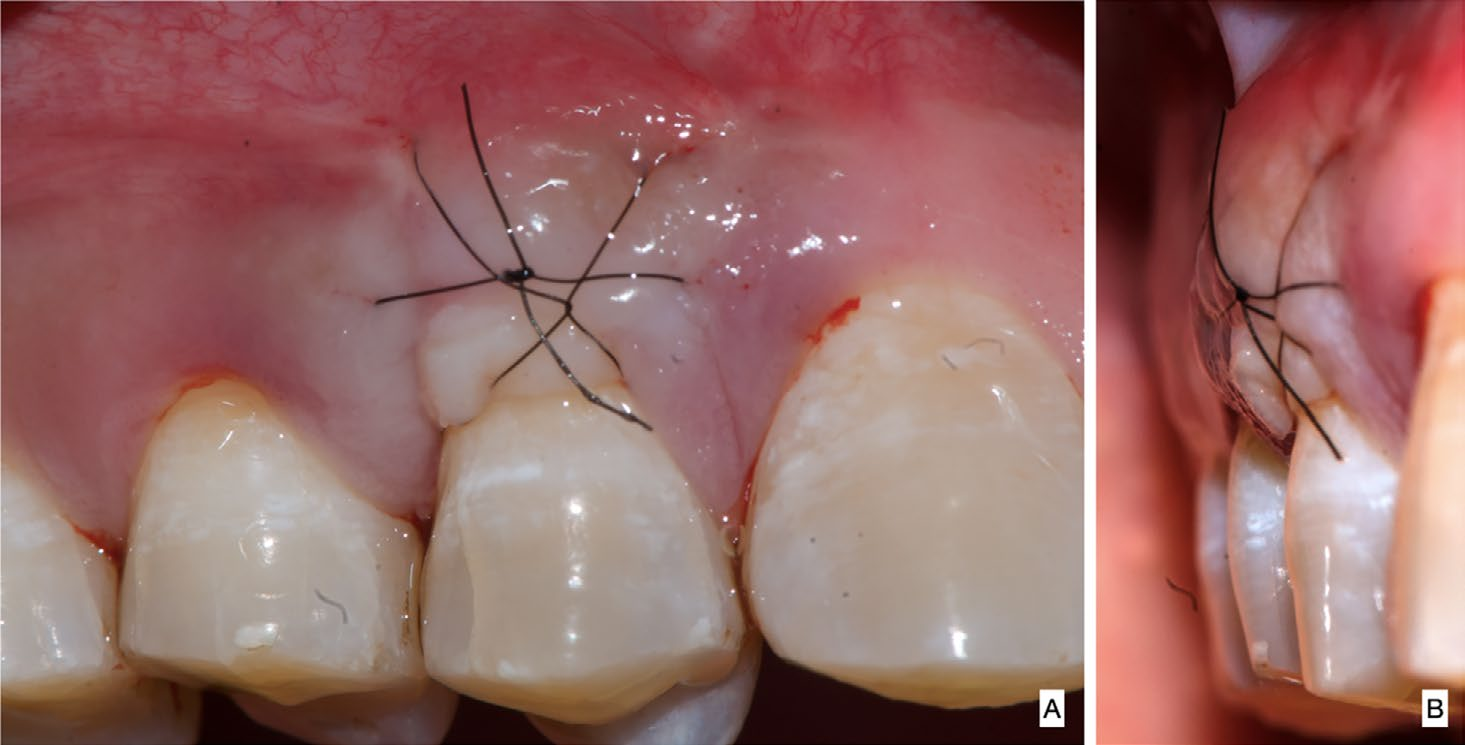
(A) CTG graft secured in the recipient bed in facial view, (B) CTG graft secured in the recipient bed in proximal view.

Following profound local anesthesia with infiltration (2% Lidocaine with 1:100,000 Epinephrine), the patient was treated for correction of gingival cleft and recession coverage in tooth number #14. The surgical technique was performed utilizing a microsurgical scalpel (SB004, MJK instruments, France.). The surgical procedure utilized in this particular case was a Dufourmentel flap, which was adapted from plastic and reconstructive surgery [5]. For the ease of understanding of the flap design, a line diagram with markings is presented (Figure 2). Free marginal gingiva, along with the white gingival cleft, was de‐epithelialized using a Castroviejo blade extending from one CEJ point angle C to D (Figure 2). For optimal edge‐to‐edge tissue adaptation, the blade was inserted at a right angle to the tissue to achieve a clear butt angle. An intrasulcular incision was placed to connect the two point angles. Subsequently, an oblique incision of split thickness was made, commencing from the area of the gingival cleft and extending beyond the mucogingival junction (MGJ). An analogous incision was made, commencing at point angle C, and it was further extended in order to gain access to the alveolar mucosa (Figure 3). A partial thickness incision, denoted as AE, is made by dividing the angle obtained by extending the lines DA and BA in half. Beyond the MGJ, the incision is extended to incorporate the de‐epithelialized white cleft, thereby maximizing the mobility of alveolar mucosa, which facilitates passive flap advancement. The second partial thickness incision CF is positioned at a right angle to the line extending from AE. The modified procedure begins at point C, which is the intersection of CEJ and the facial line angle of the tooth. The length of the incision CF is 3 mm longer than the breadth of the recession, DC, in order to include the measurements of the initially de‐epithelialized marginal gingival tissue. The incision was made within the attached gingiva for a total of 7 mm, and it was extended to a length of 10 mm at an angle of 110 degrees beyond the MGJ, denoted as FG, where G is the fulcrum point. The attainment of a 25‐degree rotation facilitates primary closure. The radius of this flap is determined by the distance between the fulcrum point G and the edge of the defect C, which produces an arc of transposition. The optimal pivot or fulcrum point should have a magnitude of 2.0 to 2.5 times the size of the defect. Thus, a partial thickness flap was created that was transposed to the adjacent recession defect. For the closure, point C transposes to D, point F to C, and G translates to F utilizing the mobility of alveolar mucosa. The transpositional flap was secured with “coaptation sutures,” which consisted of multiple single interrupted 8/0 polyglactin suture (Absorbable surgical suture, Vicryl, Ethicon, Aurangabad, India.) on each side of the flap. Passively, these coaptation sutures moved the flap margin by 1 mm coronal to the CEJ (Figure 4). The patient was prescribed oral analgesic Ibuprofen 400 mg 3 times a day for 3 days, along with 0.12% chlorhexidine for chemical plaque control. Sutures were removed one week after the surgery. After a period of 6 months, it became apparent that the white gingival cleft had been completely corrected and there was an augmentation in the WKT. The gingival examination, on the other hand, identified a residual MTR of 1.5 mm with a KTW of 3 mm (Figure 5). Consequently, a minimally invasive tunneled connective tissue grafting procedure was planned. Using a microsurgical knife (SB002, MJK instruments, France.), tunneling was done to create a supra‐periosteal mucosal tunnel (Figure 6). The subepithelial connective tissue graft (CTG) was obtained with an epithelial collar of 2 mm thickness and a remaining connective tissue graft thickness of 1 mm (Figure 7A,B). CTG was secured in place with a horizontal mattress suture using 6/0 polyamide* suture, leaving the epithelial collar exposed below the flap margin. By employing a horizontal cross mattress suture, the dead space between the exposed root surface, secured CTG, and overlying flap was eliminated (Figure 8A,B). Sutures were removed two weeks postoperatively. The patient received periodontal treatment with supportive measures and was assigned recall appointments every 6 months.

## 4 Conclusion and Results

**FIGURE 9:**
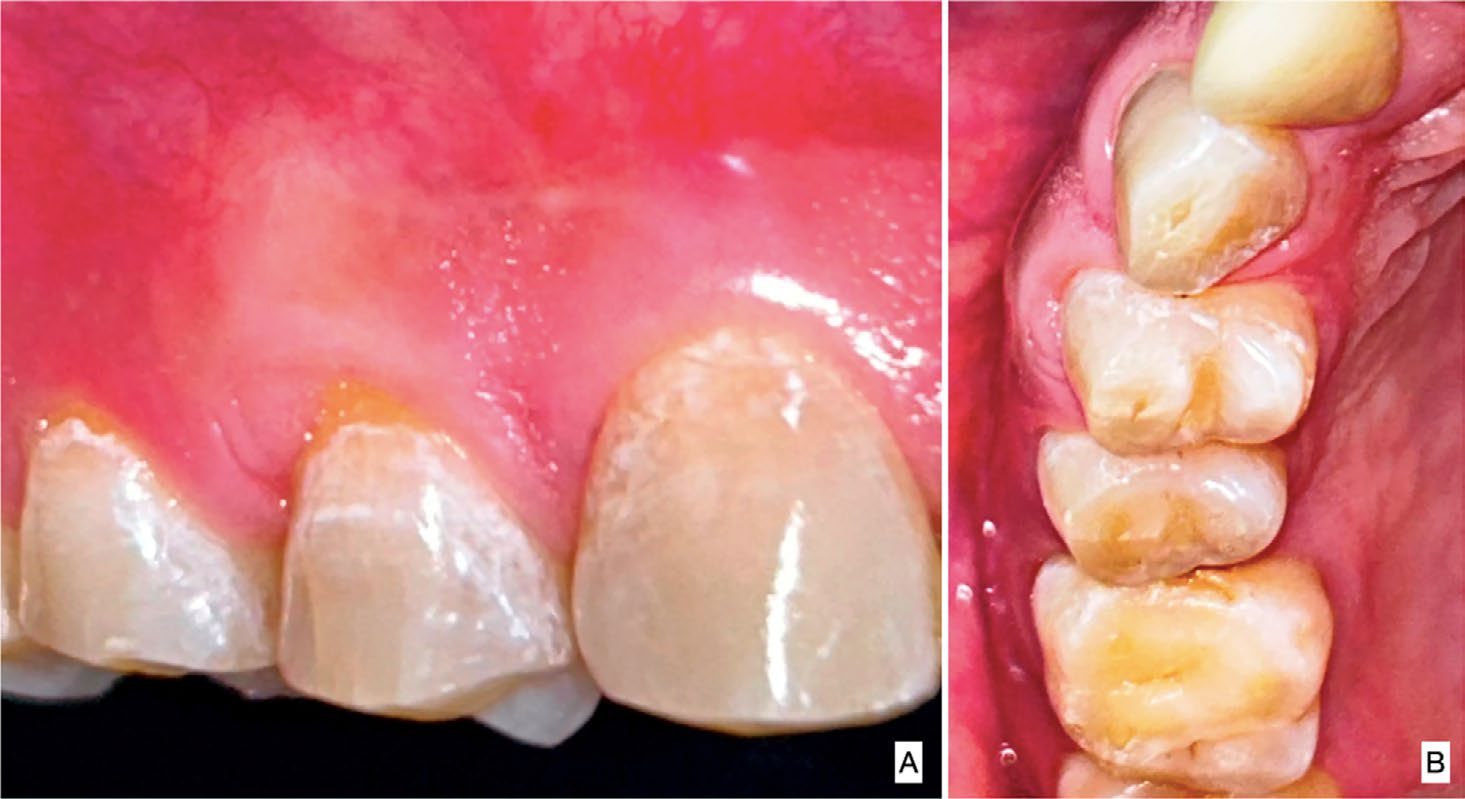
(A) 6 month postoperative follow‐up in facial view, (B) occlusal view.

**FIGURE 10:**
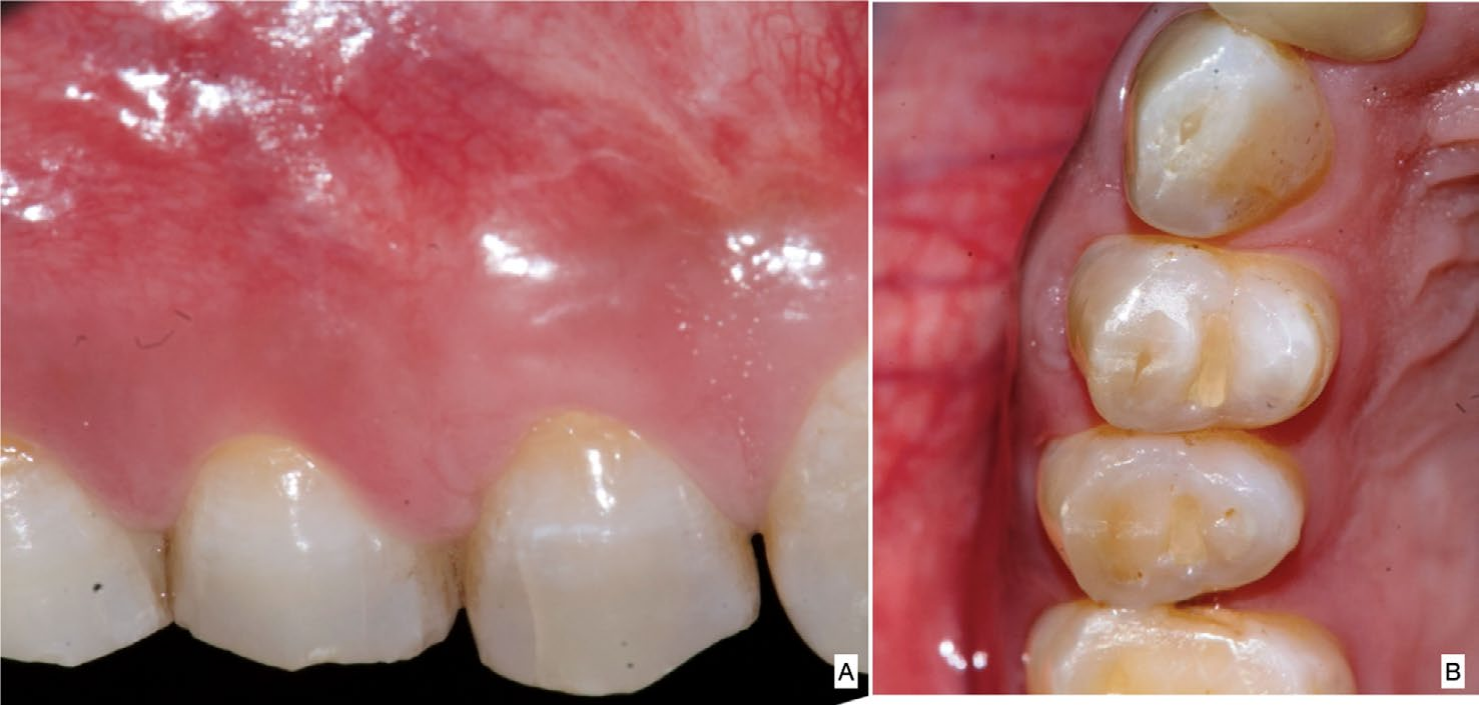
(A) 2 years postoperative follow up in facial view, (B) occlusal view.

There were no intra and postoperative complications. The Numerical Rating Scale (NRS) pain score was 2 out of 10 within the first 24 h after the first microsurgical procedure. The gingival cleft was completely eliminated along with an increase in WKT from 2 to 4 mm and WAG from 1 to 3 mm. Soft tissue phenotype remained “thick” with stable gingival margins during the six‐monthly follow‐up (Figure 9A,B) and up to 2 years (Figure 10A,B). Correction of residual recession with tunneled CTG further enhanced the soft tissue profile in terms of WKT and soft tissue phenotype (Table 1). The patient demonstrated substantial improvement in self‐performed plaque control measures. A two‐year follow‐up revealed that the gingival margin was stable and was maintained coronal to CEJ.

**TABLE 1:** Clinical parameters of the reported case.

	Baseline	6 months postoperative	12 months	24 months
RH	3 mm	1.5 mm	0	0
RW	3 mm	3 mm	0	0
CAL	5 mm	3 mm	0	0
WAG	1 mm	1.5 mm	3 mm	3 mm
KTW	2 mm	3 mm	4 mm	4 mm

## 5 Discussion

Gingival clefts are inconspicuous lesions, necessitating early diagnosis in order to prevent epithelial tissue breakdown and future recession. Owing to their unobtrusive nature, it is preferred that the treatment is undertaken using the operating microscope in order to ensure complete de‐epithelialization of the white cleft followed by ideal coaptation of the tissues. Microsurgical principles were employed in the management of gingival cleft closure in view of benefits in terms of reduced postoperative morbidity and better patient‐reported outcomes. Undoubtedly, it improves the manipulation of soft tissue, ensures perfect closure, and accelerates the healing process [6].

**FIGURE 11:**
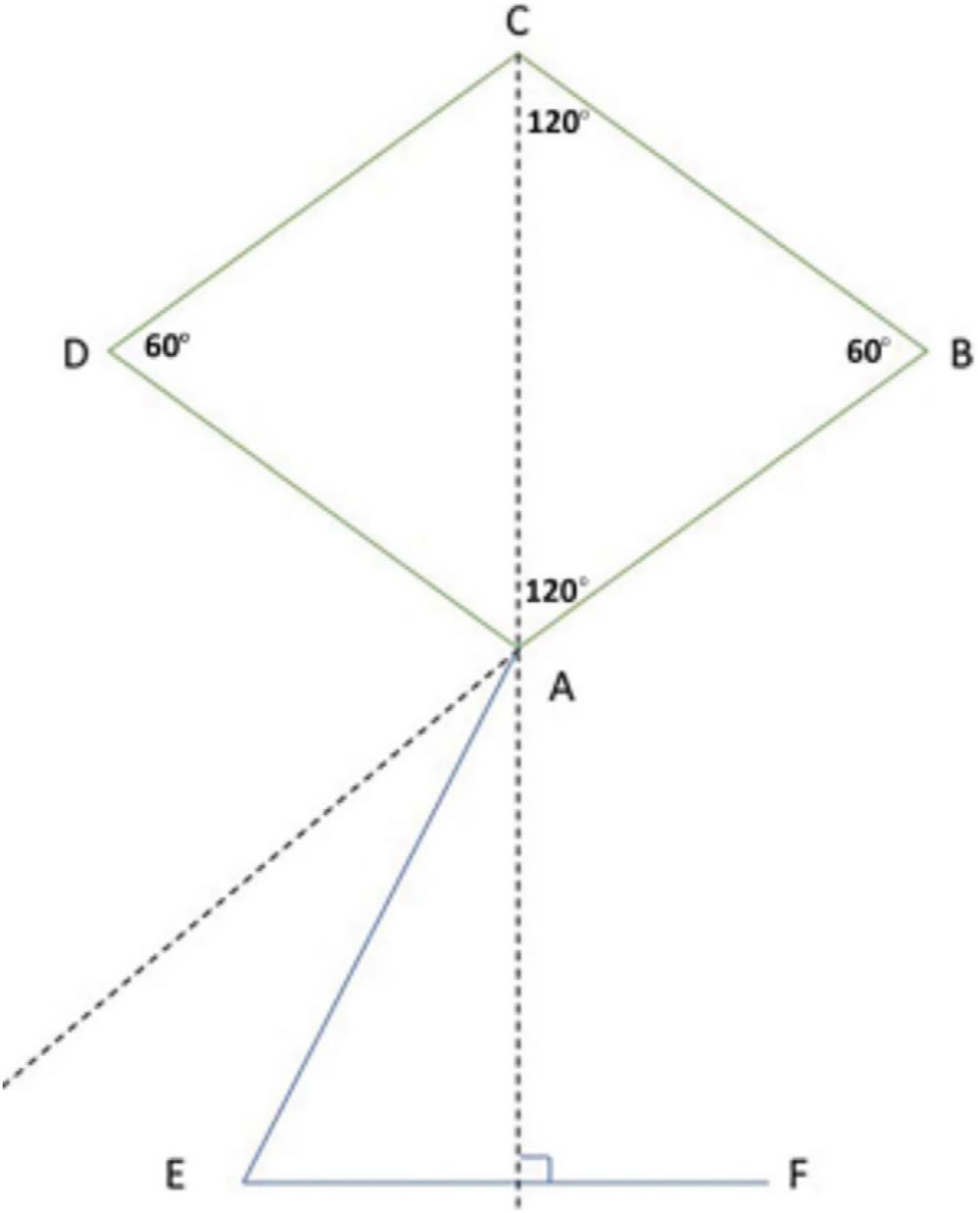
Geometric design of Dufourmentel flap.

**FIGURE 12:**
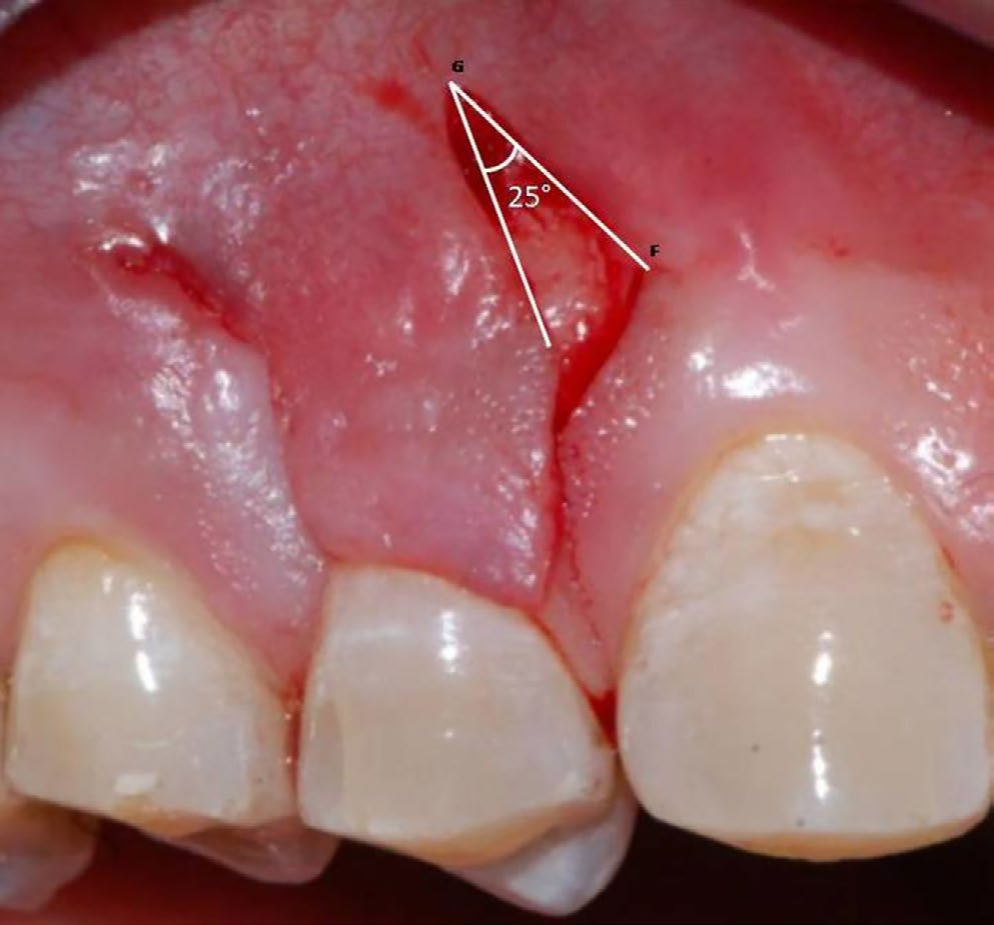
25 degree flap rotation of the Dufourmental flap.

The transposition flap is a random pattern flap that involves the movement of tissue across an intervening segment of tissue [7]. The design ensures that the donor site location is positioned next to the defect. The “Dufourmentel flap” is a type of flap used in cosmetic and reconstructive procedures. It achieves primary closure by adhering to specific geometric principles, which result in a broader pedicle base and enhanced blood supply [5]. The technique is employed mostly in scalp reconstruction, involving a rhombus with equal sides around the affected area, with angles of 60 degrees and 120 degrees [7]. The rhombus flap design, which is derived from the Dufourmentel flap, is illustrated in Figures 11 and 12. The points ABCD are labeled to facilitate comprehension. The initial incision, AE, divides the angle created by extending AB and AC into two equal parts, and its length is equivalent to the sides of the rhombus. The second incision, EF, is perpendicular to the extended line AC and is also of identical length. Point A will undergo rotation to reach point C, point E will undergo rotation to reach point D, and point F will undergo translation to reach point A.

The principles of flap design used in the Dufourmentel flap, which is commonly performed in plastic surgery, were applied to periodontal surgery for the first time in order to accomplish complete root coverage in this particular case. The recession site is shaped like a rhomboid, with designated spots labeled as A, B, C, and D (Figure 2). The modified procedure begins at point C, which is the intersection of the CEJ and the facial line angle of the tooth [8]. The arc of rotation should not exceed 30 degrees [9] otherwise, it leads to “Burrow's triangle,” also known as a ‘standing cone deformity’. This deformity can also be observed in most periodontal reconstructive surgeries as a fold of tissue at the ends of incision lines. Point A serves as a promontory, along which the flap, whose leading edge is shared by the defect margin, is mobilized and translated over the defect. To achieve the primary closure of both the recipient and donor sites, both the promontory (A) and pivot points (G) were moved by this modified Dufourmentel flap. This particular flap design aided in complete closure of the cleft, and MTR was addressed with minimally invasive tunneled CTG [10].

CTG as an autogenous graft is still considered to be the gold standard in periodontal plastic microsurgical procedures and induces keratinization of the overlying epithelium [11]. The use of CTG is considered to achieve RH reduction and also enhance gingival margin stability and aesthetic outcomes [12]. CTG with epithelial collar was employed in the present case in order to achieve graft placement 1 mm beyond the CEJ, which further eliminated the need of coronal advancement of the overlying tunneled flap at second stage surgery. Six months postoperative evaluation revealed better soft tissue profile in terms of improved WAG and WKT. The epithelial collar might have contributed to KW gain during the initial stage of healing [13]. Both CTG techniques (with or without the epithelial collar) have been shown to provide predictable and successful root coverage outcomes [13]. The predictability of this technique was enhanced using the microsurgical concept, since it offered better vascularization of the subepithelial CTG in root coverage compared to a traditional macrosurgical approach. Because of dual vascularity and precise incisions, the risk of necrosis of the CTG is minimized, which aids in enhanced wound healing [14].

The importance of soft tissue phenotype (Gingival Thickness, GT) has been analyzed previously. A critical threshold flap thickness of > 1.1 mm was suggested from a systematic review concluding a positive association exists between GT and root coverage [15]. The result of the present case is in agreement with observations from the review. The thick gingival phenotype was further enhanced by the incorporation of CTG at the residual recession site, and hence a stable soft tissue profile was appreciated even after 2 years.

Gingival cleft management necessitates a comprehensive treatment approach that considers the etiopathogenesis and clinical manifestation of the condition, in addition to a meticulous diagnosis. A recent case report described the treatment of a red Stillman's cleft using subgingival curettage with area‐specific Gracey curettes to stimulate bleeding, followed by the application of sodium hyaluronate gel. Healing occurred within 3 months with ~0.5 mm recession reduction, although the evidence remains weak [16]. Such minimally invasive methods are not generally recommended for white clefts due to the presence of epithelial lining, which requires surgical excision. A modified microsurgical tunnel technique with connective tissue graft (CTG) for treating gingival recession was established where an envelope‐style split‐thickness flap and CTG were inserted beneath a minimally invasive tunnel, preserving blood supply and ensuring superior aesthetic outcomes [10]. While this technique was primarily developed for gingival recessions, the core principles—atraumatic split‐thickness tunneling, microsurgical instruments, and CTG placement were found to be highly applicable to the surgical closure of white gingival clefts. The precision and graft stabilization reduced trauma and optimized soft tissue phenotype.

In a case series, three epithelialized white gingival clefts were managed using a microsurgical, bilaminar approach with CTG, and precise cleft approximation was done under magnification. At 3‐year follow‐up, results showed complete closure, 1 mm probing depth reduction, attachment gain, increased attached gingiva (to ~3 mm), and improved tissue thickness. The microsurgical method minimized trauma and enhanced aesthetic predictability [3]. In order to obtain predictable outcomes, the treatment selection must be based on sound periodontal plastic and microsurgical principles. Additionally, postoperative patient compliance can be a critical unquantifiable factor that impacts clinical outcomes. Consequently, treatment necessitates consistent recall and maintenance. It is underscored that the effectiveness of modified Duformentel flap in the management of gingival cleft combined with MTR can only be definitively recommended following the execution of rigorous randomized controlled clinical trials to evaluate its impact.

The case report presents a successful management of an isolated white gingival cleft associated with MTR executed microsurgically. It serves as an illustration of the application of geometric flap principles from plastic surgery to the modification of the Dufourmentel flap in periodontal plastic surgery. Modification being the utilization of geometrically planned incisions extending in the alveolar mucosa in order to gain mobility of transposition flap and increase in the width of the flap by 3 mm in order to cover the de‐epithelialized marginal gingiva across the RW. Residual recession prompted a secondary intervention involving minimally invasive tunneled connective tissue grafting. This procedure further enhanced the WKT and WAG in addition to resolving the MTR. Throughout the course of treatment and during the follow‐up period, no intra or postoperative complications have been reported, demonstrating the efficacy and safety of the microsurgical technique employed in this particular case. Supportive periodontal therapy and regular recall visits were scheduled to ensure the maintenance of the periodontal health. This case emphasizes the importance of careful case selection, detailed surgical planning and precision in microsurgical execution for optimal periodontal outcomes.

## Author Contributions

**Ruchi Harish:** conceptualization, data curation, formal analysis, methodology, resources, writing – original draft. **Saravanan Sampoornam Pape Reddy:** data curation, formal analysis, investigation, methodology, visualization. **Shaili Pradhan:** project administration, supervision, validation, writing – review and editing.

## Ethics Statement

The authors have nothing to report.

## Consent

A written informed consent form was obtained from the patient reported in this case report.

## Conflicts of Interest

The authors declare no conflicts of interest.

## Data Availability

The authors have nothing to report.
